# Pediatric Reference Intervals for Free Thyroxine and Free Triiodothyronine by Equilibrium Dialysis-Liquid Chromatography-Tandem Mass Spectrometry

**DOI:** 10.4274/jcrpe.2152

**Published:** 2016-03-01

**Authors:** Sonia L. La’ulu, Kyle J. Rasmussen, Joely A. Straseski

**Affiliations:** 1 ARUP Institute for Clinical and Experimental Pathology, Salt Lake City, Utah, USA; 2 University of Utah Health Sciences Center, Department of Pathology, Salt Lake City, Utah, USA; 3 These authors contributed equally to this work.

**Keywords:** Pediatric, reference interval, free thyroxine, free triiodothyronine, mass spectrometry

## Abstract

**Objective:**

Thyroid hormone concentrations fluctuate during growth and development. To accurately diagnose thyroid disease in pediatric patients, reference intervals (RIs) should be established with appropriate age groups from an adequate number of healthy subjects using the most exact methods possible. Obtaining statistically useful numbers of healthy patients is particularly challenging for pediatric populations. The objective of this study was to determine non-parametric RIs for free thyroxine (fT4) and free triiodothyronine (fT3) using equilibrium dialysis-high performance liquid chromatography-tandem mass spectrometry with over 2200 healthy children 6 months-17 years of age.

**Methods:**

Subjects were negative for both thyroglobulin and thyroid peroxidase autoantibodies and had normal thyrotropin concentrations. The study included 2213 children (1129 boys and 1084 girls), with at least 120 subjects (average of 125) from each year of life, except for the 6 month to 1 year age group (n=96).

**Results:**

Non-parametric RIs (95th percentile) for fT4 were: 18.0-34.7 pmol/L (boys and girls, 6 months-6 years) and 14.2-25.7 pmol/L (boys and girls, 7-17 years). RIs for fT3 were: 5.8-13.1 pmol/L (girls, 6 months-6 years); 5.7-11.8 pmol/L (boys, 6 months-6 years); 5.7-10.0 pmol/L (boys and girls, 7-12 years); 4.5-8.6 pmol/L (girls, 13-17 years); and 5.2-9.4 pmol/L (boys, 13-17 years).

**Conclusion:**

Numerous significant differences were observed between pediatric age groups and previously established adult ranges. This emphasizes the need for well-characterized RIs for thyroid hormones in the pediatric population.

**WHAT IS ALREADY KNOWN ON THIS TOPIC?**

Early detection of thyroid abnormalities is critical in young children. However, the challenges of determining pediatric reference intervals (RIs) are well known. Obtaining sufficient sample numbers in healthy children is often exceedingly difficult and underscores the importance of reporting these types of studies. Free thyroxine (fT_4_) and free triiodothyronine (fT_3_) RIs have additional complexities, including accurate measurement of free hormones and the necessity of screening for subclinical thyroid disease in the reference population.

**WHAT THIS STUDY ADDS?**

This work provides nonparametric RIs for fT_4_ and fT_3_ for children from 6 months through 17 years of age. Uniquely, our study utilized a significant number of samples (n=2213) from both healthy boys (n=1131) and girls (n=1082). All individuals were screened for thyroid stimulating hormone and thyroid autoantibodies prior to inclusion in our study and had no known medical conditions or medication use. This study was performed using an in-house equilibrium dialysis-high performance liquid chromatography-tandem mass spectrometry method. We point out the importance of determining method-specific intervals, which has been recommended, particularly for fT^4^. This work will be useful for any laboratory or clinician serving a pediatric population.

## INTRODUCTION

Identifying thyroid dysfunction is critical in young children. An imbalance in thyroid hormone concentrations early in life can have long-term ramifications, such as developmental delays, mental and/or growth retardation. Numerous conditions affecting the thyroid gland in children and adolescents may result in thyroid dysfunction. Primary congenital hypothyroidism is one of the more common thyroid abnormalities that can occur in children, with a prevalence of 1 in 3000 infants (1). It results when the thyroid gland is unable to produce sufficient amounts of thyroxine (T_4_) or triiodothyronine (T_3_). Graves’ disease is the most common cause of hyperthyroidism in children, and although rare, can be fatal if not properly diagnosed and treated (2).

Symptoms of thyroid disease are not always obvious in healthy populations, therefore, laboratory measurements are of increased value. Thyroid stimulating hormone (TSH) is the initial screening test for assessment of thyroid dysfunction. The dynamic equilibrium that exists between free and protein-bound forms of T_3_ and T_4_ hormones makes the assessment of their in vivo concentrations complex. Free thyroxine (fT_4_) and free triiodothyronine (fT_3_) are analyzed when additional diagnostic information is needed in patients with suspected thyroid disease. Identifying the presence of thyroglobulin autoantibodies (TgAb) or thyroid peroxidase autoantibodies (TPOAb) is also useful in detecting autoimmune disorders that affect thyroid function (3).

Establishing reference intervals (RI) for thyroid function tests in healthy pediatric subjects is essential to effectively diagnose disease in this patient population. The National Academy of Clinical Biochemistry recommends obtaining method-specific RIs for thyroid hormones, particularly for fT_4_ (4). Mass spectrometry is considered a more specific method overall, particularly in challenging populations and situations requiring accurate measurement of small concentrations. Separation techniques such as equilibrium dialysis are useful for obtaining more accurate and consistent results in cases where alterations in thyroid hormone binding protein concentrations are suspected (4).

Getting access to samples from truly healthy individuals, particularly children, is often complicated. Challenges include obtaining consent, defining the healthy status in various stages of childhood development, restrictions imposed by institutional review boards, and small sample volumes due to maximum blood draw limits. These complexities often lead to small data sets that lack statistical power. The samples included in this study were from a well-characterized repository (CHILDx^®^ program) that included sufficient information to confirm the health status of over 6000 children in total.

The purpose of this study was to improve the clinical utility of diagnostic tools available to physicians working with pediatric patients suspected of thyroid disease. Non-parametric RIs were established for fT_4_ and fT_3_ by equilibrium dialysis-high performance liquid chromatography-tandem mass spectrometry (ED-LC-MS/MS) using a well-characterized, healthy population of children ages 6 months-17 years (n=2213). Importantly, the population used for these determinations had normal TSH concentrations, lacked thyroid autoantibodies, and were of sufficient sample size to apply non-parametric statistics.

## METHODS

### Sample Acquisition

Samples used for this study were part of the CHILDx® repository of samples from healthy children. Two different approaches were used for sample acquisition. Children 6 months-6 years of age were assessed for enrollment by a physician assistant at Primary Children’s Medical Center (Salt Lake City, Utah) prior to elective, non-invasive, outpatient surgery, such as dental surgery, umbilical hernia repairs, nevus removals, orchiopexies, or orthopedic procedures. No unhealthy, medicated, or inpatient children were enrolled. Blood was drawn while patients were under gas anesthesia but prior to general anesthesia administered by IV. For children 7-17 years of age, subjects volunteered to participate and were recruited by institutional review board (IRB)-approved flyers, advertisements in magazines, and by word of mouth. An evaluation was conducted that included a full physical examination followed by blood and urine collection. Subjects were excluded for known medical conditions, medication use (other than seasonal allergy medication), or had a medical history that would consider them to be unhealthy. Eligible subjects from both age groups were enrolled after obtaining parental permission. All subject enrollment and testing protocols were approved by the University of Utah IRB.

### Sample Processing and Testing

Blood was drawn into serum separator tubes and allowed to coagulate for 30 minutes prior to centrifugation. All samples were aliquoted and cryogenically frozen. In an effort to include only subjects with normal thyroid function, all samples were tested for TgAb and TPOAb on the ARCHITECT i2000SR (Abbott Diagnostics, Abbott Park, IL). Upper reference limits of 14.4 IU/mL for TgAb and 3.9 IU/mL for TPOAb were determined previously (5). Autoantibody negative samples were then tested for TSH (MODULAR ANALYTICS E170, Roche Diagnostics, Indianapolis, IN) to establish TSH RIs specific for our population. The central 95% non-parametric RI was established for TSH and samples outside of these ranges were excluded from fT_4_ and fT_3_ testing. Analysis by ED-LC-MS/MS for fT_4_ and fT_3_ was performed as previously described (6). Briefly, serum samples were dialyzed 1:1 against a simple protein-free buffer for 20 hours at 37 °C. Thyroid hormones in dialysates were purified by online solid-phase extraction, then chromatographically separated and quantified in positive ion and multiple reaction monitoring modes. Total imprecision was reported to be <10%. Adult non-parametric fT_4_ and fT_3_ RIs were previously established as 16.5 (95% confidence interval (CI), <15.1 to 17.5) to 28.6 (26.1 to >36.9) pmol/L for fT4 and 5.6 (95% CI, <4.8 to 6.4) to 10.4 (95% CI, 10.2 to >10.9) pmol/L for fT3 (n=67 females, n=70 males) (6).

### Statistical Analysis

Non-parametric RIs were determined using EP Evaluator software (Data Innovations, South Burlington, VT). Differences between ages or gender were first identified by determining whether the determined reference limits were contained within the 95% CIs of the adjacent group or other gender. If limits were contained within the adjacent CI, age groups and genders were combined. Statistical significance of the resulting partitions was confirmed by calculating p-values using GraphPad Prism (GraphPad Software, San Diego, CA). Pediatric reference limits were compared to adult CIs to determine whether ranges were different. Dixon’s test was used to identify and remove outliers.

## RESULTS

A total of 2,540 subjects were initially evaluated for this study. Subjects with thyroid autoantibody concentrations above the established thresholds (n=172) and TSH concentrations outside the determined RIs (n=155) were excluded from RI analysis for fT_4_ and fT_3_. The central 95% non-parametric RIs for TSH, as determined using the E170 (n=2284), are provided (Table 1). Subjects that were negative for thyroid autoantibodies, within the central 95% of the established RI for TSH, and had sufficient volume (n=2213) were analyzed for fT_4_ and fT_3_ using ED-LC-MS/MS. More than 120 samples (average n=125) were tested for fT_4_ and fT_3_ from each year of life, with the exception of the 6 months-1 year age group (n=96).

**Figure 1:**
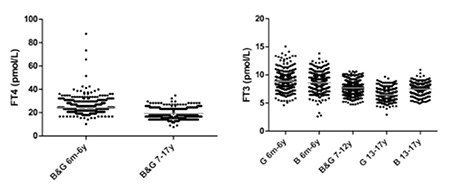
Scatter dot plot representation of the distribution of free thyroxine and free triiodothyronine in children ages 6 months to 17 years. Age groups and genders were combined when no significant differences were observed. The gray line represents the mean. FT4: free thyroxine, fT3: free triiodothyronine, B: boys, G: girls, m: months, y: years

The established pediatric RIs for fT_4_ are summarized in Table 2 along with a dot plot of the data (Figure 1). FT_4_ concentrations ranged from 7.7 to 87.7 pmol/L (median=20.6 pmol/L). No significant differences were observed when partitioning by gender for fT_4_, therefore, genders were combined. Only differences between the 6 months-6 years and the 7-17 years of age groups were statistically significant and warranted partitioning (p-value <0.0001). Pediatric ranges were compared with RI previously established for adults (see Methods and reference 6) and were considered different if limits did not fall within the 95% CIs of the adult reference limits. For fT_4_, there were differences between adult values and both age groups for the lower and upper limits. The lower limit for the 6 months-6 years of age group was higher than that for adults (designated “H” in Table 2), while that value for the 7-17 year old age group was lower (designated “L” in Table 2). The fT_4_ upper limit was different only in the 7-17 year olds and found to be lower than that in adults.

The established pediatric RIs for fT_3_ are provided (Table 2, Figure 1). FT_3_ concentrations ranged from 2.6 to 15.1 pmol/L (median=7.9 pmol/L). Due to significant differences, genders were partitioned for the 6 months-6 years (p-value, 0.038) and 13-17 years age groups (p-value <0.0001). Statistically significant differences were also observed between age groups 6 months-6 years, 7-12 years, and 13-17 years (p-values <0.0001 to 0.002). Within the 6 months-6 years age group, upper limits were higher in girls than boys. In contrast, upper limits were higher for boys than girls in the oldest age group (13-17 year olds). Upper limits were higher for ages 6 months-6 years than for all other age groups. When compared to adult RI, the only difference observed for the fT_3_ lower limit was for 13-17 year old girls being lower than that for adults. However, for the fT_3_ upper limit, all of the pediatric age groups were different than the adult population; 6 months-6 year olds (both boys and girls) were higher and both of the older age groups were lower than the adult range.

## DISCUSSION

Thyroid dysfunction during childhood development may result in serious outcomes, including mental impairment and growth delays. Early diagnosis allows for rapid intervention that can almost entirely reverse symptoms. This study was performed to improve clinicians’ ability to correctly diagnose thyroid disorders in children by providing population-specific RIs. Providing accurate RIs requires testing of well-characterized, healthy patients from a sample set of adequate size to provide statistical relevance. These criteria are particularly difficult to meet in pediatric populations; however, we were able to address both in this study.

TSH results are often used as the primary indicator for assessing thyroid dysfunction and may be followed by fT_4_ and fT_3_ testing when TSH results are close to reference limits or if further evidence of thyroid disease is required. Even though our main focus was to establish RIs for fT_4_ and fT_3_, those results could be skewed by including children with unapparent thyroid disorders. Because symptoms of thyroid disease are not always recognized or diagnosed, we screened samples and only included those that were thyroid autoantibody negative and were within the determined TSH RI.

Thyroid hormone concentrations are highest immediately following birth (7). TSH concentrations rise in response to the temperature shock of leaving the in utero environment, which in turn increases the concentrations of T_4_ and T_3_. Higher TSH concentrations are also expected in children due to progressive maturation and modulation of the hypothalamic-pituitary-thyroid axis during development (8). Expectedly, the TSH RIs that were established for children in this study were higher than the recommended range for adults (0.3-3.0 mIU/L) (4) and gradually decreased with age.

Initial partitioning of gender and age groups showed no significant differences for fT_4_; therefore, genders and most age groups were combined. In contrast, the fT_3_ RIs showed significant differences between age and gender and more partitions were required. Differences were particularly notable in girls where the difference between upper limits of girls’ ages 6 months-6 years and girls’ ages 13-17 years was 34%. There are conflicting data regarding gender differences for fT_3_ in pediatric populations. Similar to our findings, the decline of fT_3_ in girls during puberty has been observed previously, whereas concentrations in boys remained seemingly constant (9,10,11). Kapelari et al (12) also established gender-specific RIs for fT_3_ and did not report significant gender differences for fT_4_ using the ADVIA Centaur. However, Hübner et al (13) observed gender differences for fT_3_ only within the 11-14 age group, which is in contrast to ours and other studies where no statistically significant differences were observed between genders for the 13-17 year age group. Moreover, differences between boys and girls for the upper limit of fT_3_ were not reported previously by Soldin et al (14) using similar methodology to our study. The differences described here may be attributed to known differences in thyroid hormone concentrations among populations and/or methods (15,16). Of note, we have previously observed ethnic differences in thyroid hormones in pregnant individuals (17,18), and ethnicity has not been addressed in these pediatric populations. Furthermore, either lower limits, upper limits, or both, from children for fT_4_ and fT_3_ were significantly different from adult RIs (6), for every age group. The above comparisons and the numerous significant differences observed further emphasize the necessity of establishing reference ranges specific to pediatric populations.

Soldin et al (14) performed testing on pediatric subjects by LC-MS/MS using an ultrafiltration method rather than equilibrium dialysis for isolation of free hormones. For fT_4_, our RIs using ED were comparable to those determined by Soldin et al (14) using ultrafiltration performed at 37 °C. Our lower reference limits for fT_3_ were higher than the limits determined by ultrafiltration performed at both 37 °C and 25 °C. The upper reference limits showed some similarities for fT_3_ with the exception of our younger age groups being higher.

In comparison to a candidate international reference method, our ED-LC-MS/MS method demonstrated a positive bias for both fT_4_ and fT_3_ (19). Until standardization efforts of free thyroid hormone assays/methods are established, caution should be used when interpreting RIs. Laboratories need to verify RIs specific to the method and population they are using and evaluating, particularly for thyroid function testing.

A limitation of these RIs is that all samples were taken from patients living in the region surrounding Salt Lake City, UT. This resulted in a less diverse population (96% Caucasian). Due to IRB limitations, children under 6 months of age were excluded. Although thyroid testing is part of most newborn screening programs, additional studies would be needed to determine RIs for these analytes using this ED-LC-MS/MS method in preterm and newborn populations.

Detecting thyroid dysfunction early in development is critical to reversing long-term symptoms. This study provides useful RIs established from over 2200 healthy children using equilibrium dialysis and mass spectrometry, the preferred methods of analysis for fT_4_ and fT_3_ hormones. Having RIs for this specific patient population improves the ability of physicians to properly diagnose and treat children suspected of these often reversible thyroid abnormalities.

## Figures and Tables

**Table 1:**
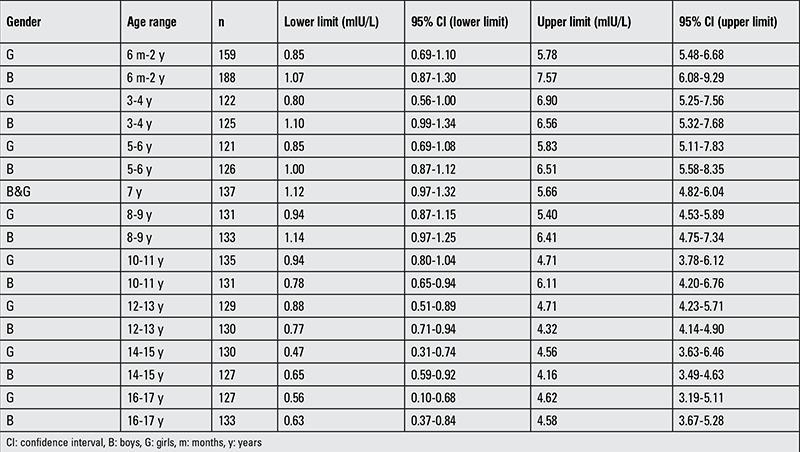
Pediatric reference intervals for thyroid stimulating hormone using the Roche E170

**Table 2:**
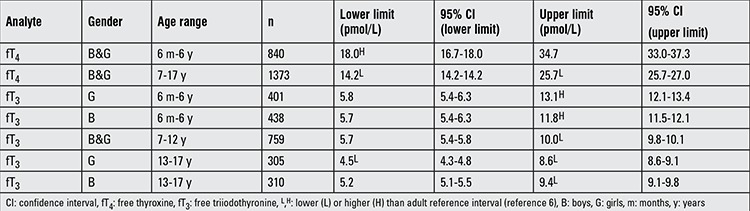
Pediatric reference intervals for free thyroxine and free triiodothyronine using equilibrium dialysis-high performance liquid chromatography-tandem mass spectrometry
